# Predicting the vertical density structure of oceanic gravity current intrusions

**DOI:** 10.1038/s41598-024-60878-x

**Published:** 2024-05-04

**Authors:** Sévan Rétif, Maria Eletta Negretti, Achim Wirth

**Affiliations:** CNRS, Grenoble INP, LEGI, Univ. Grenoble Alpes, 38000 Grenoble, France

**Keywords:** Physical oceanography, Fluid dynamics

## Abstract

Understanding the dynamics and structures in the deep ocean is one of the remaining challenges in oceanography and climate sciences. We present results from large-scale laboratory experiments of rotating down-slope gravity currents intruding into a two-layer stratified ambient, performed in the largest rotating tank in the world, the Coriolis Rotating Platform in Grenoble. By means of velocity and density measurements, we show that no mixing occurs once the current has detached from the boundary. The shape of the vertical density profile in the stratified receiving ambient enables to identify two distinct regimes: the first issued by laminar transport through Ekman dynamics, the second by turbulent transport due to intermittent dense water cascading. Vertical density gradients reveal a piece-wise linear dependence on the density anomaly for the turbulent transport, suggesting an advection-diffusion process. For the turbulent regime, the scale height is deduced and an analytical model based on the critical Froude number is proposed to predict its value. Results show that the total thickness of the intruding current is on average 2.5 times the scale height. For laminar intrusions the scale height diverges whereas the thickness of the intrusion is a few times the Ekman layer thickness. Comparing the intrusion scale height with its measured vertical extension has led to a criteria to distinguish between laminar and turbulent regimes, which is corroborated by two additional independent criteria, one based on the sign of the local vorticity and the other based on the local maxima of the vertical density gradient. The model allows us to connect laboratory experiments to deep sea observations, gravity currents and Meddies and emphasizes the importance of laboratory experiments in understanding climate dynamics.

## Introduction

Ocean circulation is one of the most affecting dynamics for the Earth’s climate system, because of the huge amount of heat and CO_2_ stored in, and transported by, the ocean and also exchanged with the atmosphere and the cryosphere, see i.e. the sixth IPCC report^1^. The global circulation is driven by wind forcing at the surface and influenced by buoyancy and the Coriolis force. While near surface ocean circulation is well observed, understood and modeled, deep ocean dynamics is remote and enigmatic^2,3^. Ocean gravity currents are known to transport salty and cold water from a coastal environment towards the deeper ocean. They are up to 20 km large and 200 m thick^4^ and have been observed in many places around the world. An example is the strait of Gibraltar, where saltier water from the Mediterranean reaches the Atlantic ocean water of lower salinity, creating the Mediterranean outflow^5^. Other examples are: The Bab el Mandeb strait at the exit of the Red Sea^6^, the strait of Hormouz at the exit of the Persian Gulf^7^ and the Denmark strait overflow^8^. The same mechanism has been observed where the density anomaly is ensured by temperature differences, for example at the Filcimer Ice Shelf outflow into the Weddell Sea^9^. In their pioneering work, Cooper and Vaux^10^ showed that cooling by surface radiation and evaporation during winter is responsible for a heavy water mass descending down a continental slope to a depth of several hundred meters. This phenomena was observed in the Celtic Sea and in the English Channel and expected to be seen widely, especially in winter at shallow banks. These currents have an impact on the concentration of nutrients and iron in the Celtic Sea. The importance of geostrophic balance in the case of down-slope gravity currents is raised in Gill^11^, discussing a larger scale gravity current in the Weddell Sea.

A well established scheme is proposed by Shapiro and Hill^12^, where the gravity current is geostrophically adjusted along-slope within a day, while due to friction, part of this dense water mass is transported downslope. Two mechanisms are pointed out for describing this downslope transport: a near slope Ekman balance between friction, Coriolis force and buoyancy, and an Ekman drainage due to upper-layer friction. Stream-tubes models are derived^13^, in which it is assumed that physical properties vary in the stream-wise direction of the gravity current, while cross-stream variations are integrated over the cross section of the gravity current. The downstream position, thickness and density anomaly of the stream tube is calculated based on physical laws and parametrisations of the unresolved processes. These models are able to include parameterised turbulent fluxes such as entrainment, mixing, detrainment, and turbulent friction. Wirth^4^, Wåhlin and Walin^14^ as well as Ungarish^15^ suggested instead to consider the rotating gravity current as composed by two interacting parts: the main flow, the “vein”, geostrophically adjusted moving along slope at the geostrophic velocity^16^, and a friction layer in which the geostrophic balance is perturbed by friction, non linearity and turbulent fluxes. Therefore, part of the water mass escapes the vein and feeds a friction layer. All these approaches are based on a continuous down-slope evolution of the gravity current, a property which is here challenged based on laboratory experiments.

Several studies have been carried out in order to characterise the mixing involved in geophysical flows, see the review of Legg et al.^17^, but it still remains a challenging task. Considering the vertical stratification of the ocean interior Munk and Wunsch^18,19^ assumed a balance between vertical advection and diffusion, obtaining a vertical turbulent diffusivity of $\kappa _{z}\approx 10^{-4}$ m^2^ s^−1^. In the work of Kunze and Smith^20^, from ocean observations in three different regions (on topography, interior, surface), the diffusivity is measured and compared with a value of $10^{-4}$ m^2^ s^−1^, obtained from the steady vertical advective–diffusive balance of Munk^18^. These comparisons have shown that mixing does not occur in the interior but rather at the bottom slope, and that the topography variation can boost for the diapycnal eddy diffusivity. Van Haren and Gostiaux^21^ derived turbulent vertical eddy diffusivities, $\kappa _{z}$, and dissipation rates, $\epsilon$, above the Great Meteor Seamount in the Canary Basin. They found that tidal flows are the dominant factor influencing temperature variations in the area, but the local bottom slope played a crucial role in semi-diurnal frequency motions. When averaging over a fortnight (2-weeks period), the estimated overall mean values were found as: $\kappa _{z}=3 \pm 10^{-3}$ m^2^ s^−1^ and $\epsilon =1.5 \pm 0.7\times 10^{-7}$ W kg^−1^. The role of boundary mixing with respect to interior mixing for the ocean remains largely debated^20^. Negretti et al.^22^ measured the gravity current structure on the slope area confirming the dual components, the vein and the friction layer, and characterized the induced cyclonic vortices generated in the upper layer, as already observed by previous authors (e.g. Lane-Serff and Baines^23^). Wirth and Negretti^24^ and Negretti et al.^22^ explained the induced basin scale circulation of a down-slope gravity current and its intrusion into a two-layer configuration, using potential vorticity conservation. In the present paper we focus on the temporal evolution of the density structure issued by intruding oceanic gravity currents which, to the best of our knowledge, has not been considered before. This is astonishing as most gravity currents in the ocean intrude into the ambient when reaching their neutral buoyancy level.

We present results from the high quality and fine scale dataset collected during the experimental campaign TUBE^22^. In the experiment, dense saline water is injected on an inclined plane in the Coriolis rotating platform. Due to the Coriolis force ($f>0$), the down-slope current is deviated to the right approaching geostrophy. Bottom friction and auto-advection of momentum (non-linearity) lead to down-slope motion. When the neutral buoyancy is reached, at the halocline, the down-slope motion stops and the Coriolis force channels the injected water into a deep boundary current (hereafter DBC) along the slope. Part of the injected water propagates off-shore. This intrusion leaves an imprint in the density and vorticity structure in the ocean interior. Studying the features of this sequence, from injection to intrusion, is important to further our understanding of the thermohaline structure, circulation, oxygenation and CO_2_ sequestration in the world’s oceans. We identify two regimes for the intrusion based on the shape of the vertical density profiles and vorticity contours, then we determine the scale height^25^ using an advection–diffusion model^18^; a new model based on the critical Froude number is proposed to predict the averaged value of the scale height as a function of the experimental parameters; we end by showing that no mixing takes place in the interior but rather on the slope during the descent.

## Methods

**Figure 1:**
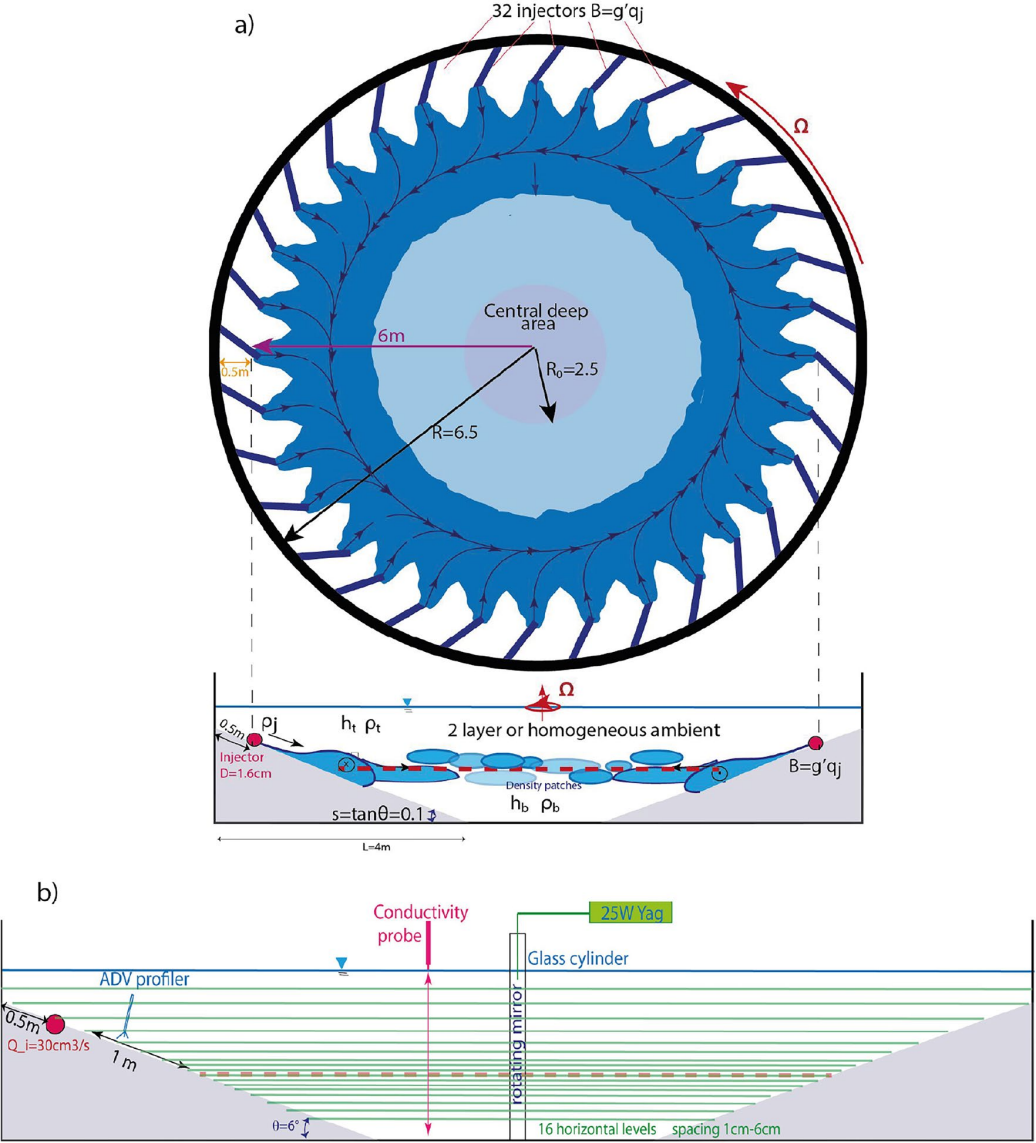
Sketch of the experimental set-up^22^. (**a**) Schematic top and side views of the experimental design. $N=32$ pipes positioned 0.5 m from the tank edge and inclined by $45^{\circ }$ inject continuously saline solutions at a density of $\rho _j$ and with a constant flow rate ranging as $q_j=32$–43 cm^3^$\textrm{s}^{-1}$ into a homogeneous or stably two-layer stratified ambient. The axisymmetric conical slope has an inclination of $\theta =6^\circ$ ($s=\tan \theta =0.1$) and a total length of 4 m. The central deep area has a diameter of 5 m. (**b**) Enlarged side view sketch showing the 16 horizontal slices for the PIV measurements, along with the ADV position and the conductivity probe mounted on a 1 m scanning axe.

The experiments have been conducted in the Coriolis Rotating Platform, the largest rotating tank in the world (13 m wide). For the experiments, the tank has a rotation period *T* and was filled with a two-layer stratification: a layer of fresh water at a density $\rho _{t}$, and a thickness $h_{t}$, above a second layer of salty water at a density $\rho _{b}$, and thickness $h_{b}$. During an experiment, at a fixed Coriolis parameter *f*, water with a density $\rho _{j}$, such that $\rho _{t}<\rho _{j}<\rho _{b}$, was injected at a constant flow rate $Q_{j}$, through 32 injectors circularly placed around the tank’s circumference just above a sloping boundary ($s=\tan \theta =0.1$) and directed at $45^{\circ }$ towards the right to accelerate geostrophic adjustment (see Fig. 1a). A vertically moving conductivity probe was used for recording vertical density profiles, every 60 s, in the central deep area (at a distance of 105 cm from the center) throughout the experiment duration (up to 220 rotational days). particle image velocimetry (PIV) is performed on 16 horizontal levels in order to record horizontal velocity data. The volume is scanned every 85 s (as seen in Fig. 1b). More details are given in the work of Negretti et al.^22^.

The relevant parameters are the slope $s=\tan \theta =0.1$ the Coriolis parameter *f*, the difference between the injected density and the density of the top layer in the receiving ambient expressed in terms of the buoyant acceleration $g'=g\frac{\rho _{j}-\rho _{t}}{\rho _{t}}$, where *g* is gravity, and the kinematic viscosity $\nu$. The characteristic velocity scale is the geostrophic velocity $V_{g}=g's/f$ and the characteristic vertical scale is given by a overall intrusion thickness $H=\gamma h_b$, where $\gamma =(\rho _j-\rho _t)/(\rho _b-\rho _t)$ measuring the portion of intruding water in the bottom layer. The characteristic horizontal scale ${\mathscr {L}}$ required for the Ekman transport $T_{Ek}=V_g\delta _{Ek}/2$, ($\delta _{Ek}=\sqrt{2\nu /f}$ is the thickness of the Ekman layer) to transport the total injected flux $Q_j$ downward, is given by the relation ${\mathscr {L}}=Q_{j}/T_{Ek}$.

The relevant non-dimensional numbers, defined with the external parameters known prior to the experiment, are: the Froude numbers in the vertical $F_{v}=V_g/(g'H)={\mathscr {O}}(10^{-1})$ and in the horizontal $F_{h}=V_g/(g'{\mathscr {L}})={\mathscr {O}}(10^{-2})$, the Rossby number $Ro=V_g/(f{\mathscr {L}})$, the Reynolds number $Re=V_g\mathscr {L}/\nu ={\mathscr {O}}(10^5)$ and the buoyancy Reynolds number $Re_b=Re F^2_h={\mathscr {O}}(10^4)$. The experimental parameters $g'$, *f*, $Q_j$, $T_{Ek}$, *H* and the values of the relevant non-dimensional numbers *Ro*, $F_v$ and $F_h$ for the different experiments considered are presented in Table 1. In the following sections, most of the results are presented for Case 1, but results from all other experiments presented in Table 1 are similar.

**Table 1:** Experimental parameters and non-dimensional numbers.

Experiment	$g'$ (cm/s^2^)	*f* (s^−1^)	$Q_j$ (l/s)	$T_{Ek}$ (m^2^/s)	*H* (cm)	*Ro* (–)	$F_v$ (–)	$F_{h}$ (–)
Case 1	5.1	0.14	1.39	6.95 × 10^−5^	0.058	1.3 × 10^−2^	0.67	0.04
Case 2	5.79	0.21	0.69	4.29 × 10^−5^	0.063	8.2 × 10^−3^	0.46	0.02
Case 3	6.09	0.21	1.19	4.51 × 10^−5^	0.042	5.2 × 10^−3^	0.57	0.02
Case 4	5.5	0.21	1.15	4.07 × 10^−5^	0.063	4.4 × 10^−3^	0.44	0.02
Case 5	5.1	0.1	1.35	1.07 × 10^−4^	0.056	3.7 × 10^−2^	0.91	0.06
Case 6	4.81	0.14	1.37	6.54 × 10^−5^	0.073	1.2 × 10^−2^	0.58	0.03
Case 7	5.5	0.21	1.36	4.07 × 10^−5^	0.052	3.7 × 10^−3^	0.49	0.02
Case 8	9.63	0.21	0.6	7.13 × 10^−5^	0.11	2.6 × 10^−2^	0.45	0.05
Case 9	7.17	0.42	0.6	1.83 × 10^−5^	0.1	1.2 × 10^−3^	0.20	0.01
Case 10	5.5	0.21	1.35	4.07 × 10^−5^	0.082	8.5 × 10^−3^	0.39	0.03
Case 11	4.51	0.21	1.11	3.34 × 10^−5^	0.073	2.2 × 10^−3^	0.37	0.01
Case 12	6	0.14	1.36	8.17 × 10^−5^	0.083	1.0 × 10^−2^	0.61	0.03
Case 13	9.16	0.21	2.54	6.78 × 10^−5^	0.106	5.6 × 10^−3^	0.44	0.02
Case 14	6.97	0.42	1	1.88 × 10^−5^	0.105	7.5 × 10^−4^	0.19	0.01
Case 15	5.3	0.05	1.2	3.14 × 10^−4^	0.076	5.1 × 10^−1^	1.60	0.23

The buoyant acceleration is defined as $g'=g\frac{\rho _{j}-\rho _{t}}{\rho _{t}}$, with *g* being the gravitational acceleration, the Coriolis parameter is $f=4\pi /T$, where *T* is the tank’s rotating period, the injection rate $Q_j$, the Ekman transport is given by $T_{Ek}=(g's/f)\delta _{Ek}/2$, where $\delta _{Ek}$ is the thickness of the Ekman layer, the Rossby number $Ro=V_g/(f\mathscr {L})$, vertical and horizontal Froude numbers $F_{v}=V_g/(g'H)$ and $F_{h}=V_g/(g'\mathscr {L})$.

## Results

### Laminar versus turbulent intrusion

**Figure 2:**
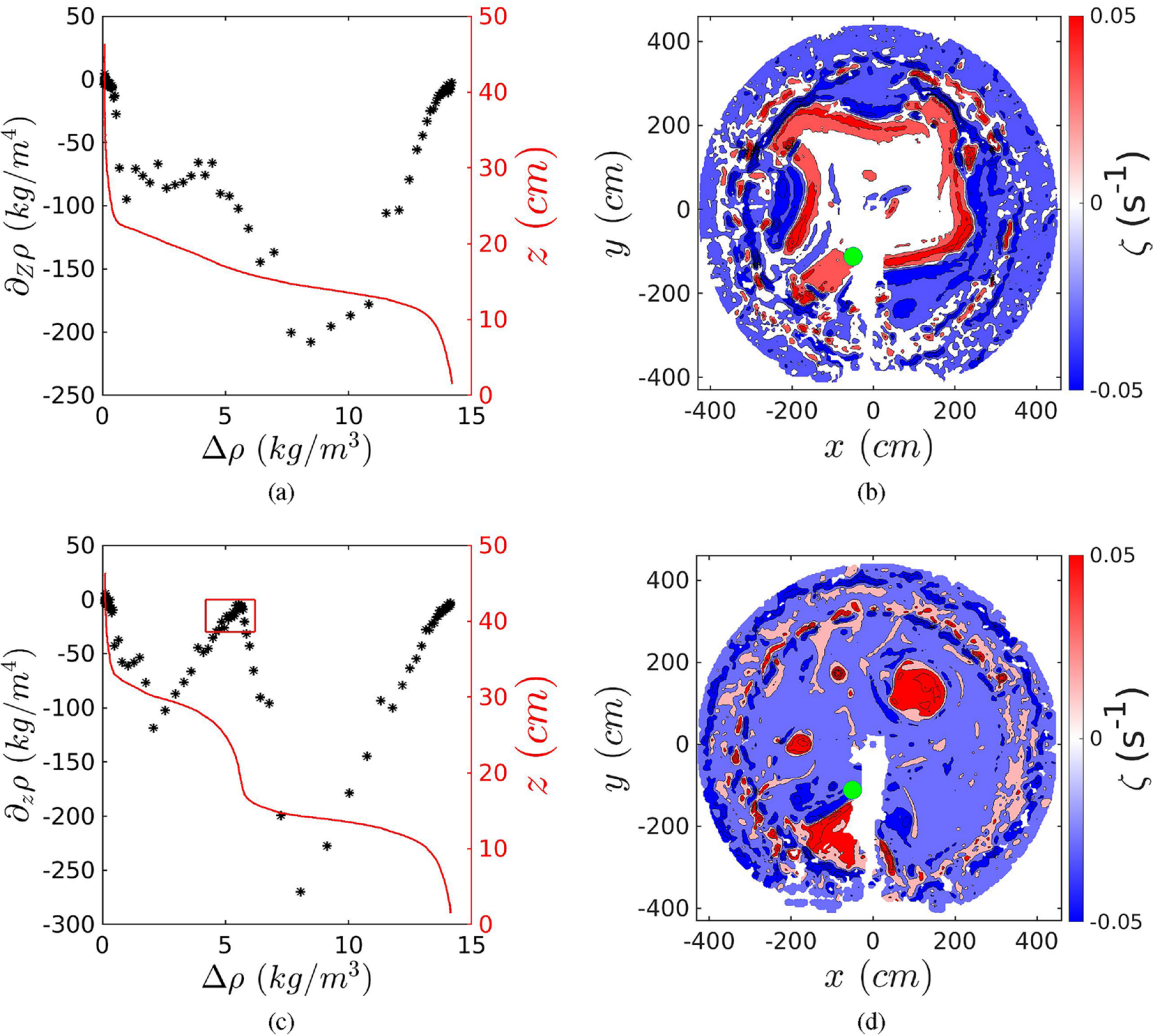
Vertical density profiles (red line), and the corresponding vertical density gradient (black stars) for $t/T=84$ (**a**) and $t/T=140$ (**c**), for Case 1. The red box in (**c**) highlights an accumulation of low values of the vertical density gradient for the local maxima criteria and it is bounded as $\rho =\rho _i\pm 1$ and $-30\le \partial _z\rho \le 0$ (**b**, **d**). In (**b**) and (**d**), horizontal vorticity fields is computed from PIV measurements at $z=15$ cm corresponding to the intrusion level, for Case 1. Green dot gives the location of the conductivity probe.

The temporal evolution of the vertical density profile allows to observe the intrusion of salty water into the initially two-layer stratified ambient at one horizontal location (cf. Fig. 1b) and to monitor its development. Figure 2a and c present the vertical density profiles (red lines) as well as the vertical density gradients (black stars) as a function of the density anomaly at different times ($t/T=84$, $t/T=140$, respectively) for Case 1. At $t/T=84$ (Fig. 2a) a zone with a linear variation of the density with depth (red curve) can be distinguished, visible as a plateau between 1 kg^3^ m^−1^ and 5 kg^3^ m^−1^ for the vertical density gradient (black stars). At $t/T=140$ (Fig. 2c), a curved shape is localised above the halocline and at density anomalies smaller than about $\Delta \rho \approx$5 kg^3^ m^−1^, leading to a double saw-tooth structure for the vertical density gradient. The corresponding vorticity field at the halocline is presented in Fig. 2b and d. At the location of the conductivity probe (Green dot in Fig. 2b,c) a positive vorticity is present at $t/T=84$ in Fig. 2b, while a negative vorticity is registered for $t/T=140$ in Fig. 2d.

Two mechanisms of intrusion are so identified: in the first, the dense water flows down the slope through laminar Ekman transport which feeds the DBC forming at the halocline. The second mechanism of intrusion, taking the form of dense water cascading, pushes the original DBC toward the central deep area, making it meander, as visible in Fig. 2b characterized by high positive vorticity values. This second mechanism of intrusion performs inertial overshoots characterised by negative relative vorticity and is visible in Fig. 2b where strong negative vorticity zones (dark blue) are located where the DBC is meandering. The horizontal dynamics and its spatio-temporal structure will be published in Tassigny et al.^26^.

At $t/T=140$ (Fig. 2d), the turbulent intrusions have broken the original DBC confining the positive vorticity to a few cyclones and are captured by the conductivity probe, which is now located in a zone where $\zeta$ is negative.

**Figure 3:**
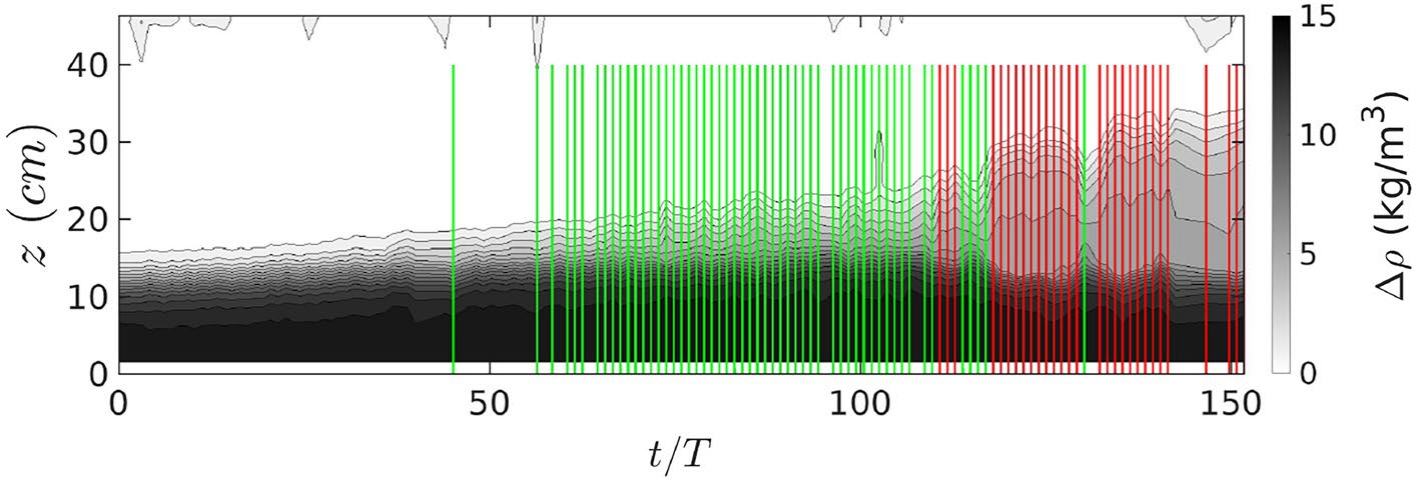
Hovmöller diagrams of the vertical density profile for Case 1. Detection of intrusion regimes: vertical green and red lines represent the detected laminar and turbulent intrusions, respectively.

The Hovmöller diagram for Case 1 is given in Fig. 3. Laminar intrusions characterised by local linearly varying vertical density profiles lead to a uniform and slow thickening of the halocline. They are highlighted by the vertical green lines in Fig. 3. Intrusions from dense water cascading, characterised by an exponential shape of the vertical density profile, on the other hand, lead to an abrupt vertical expansion of the halocline thickness, a close to homogeneous core and are highlighted by vertical red lines in Fig. 3. We observe that after periods of purely laminar intrusions, periods of purely turbulent intrusions can follow as in experiment Case 1 (Fig. 3), or turbulent intrusions can happen intermittently as observed on other experiments. No apparent correlation between the varied experimental parameters and this behavior has been found. Vorticity contours have shown that this intermittent behavior is due to the meandering shape of the DBC.

To quantitatively discriminate between the two regimes of intrusions, laminar versus turbulent, we considered three independent criteria. The first consists in detecting a pronounced local maxima in the vertical density gradient distribution at the injected density anomaly $\rho _{j}$ as shown in Fig. 2c (black stars). This local maxima criteria detects a sharp density gradient, which is translated in an accumulation of low vertical density gradient values as highlighted in Fig. 2c by the red box. The sign of the vorticity $\zeta$ measured at the probe location can also be used as a criteria. A last criteria, our principal, is based on the exponential variation of density with depth as given in Fig. 2a and c. Munk and Wunsch^18,19^ state that the vertical structure of the deep ocean is a consequence of a balance between upward advection *w* and downward diffusion $\kappa$. This approach has also been applied to smaller scale, time-dependent motion in the Adriatic^3^. It was furthermore observed through potential vorticity conservation^24^ that there is an overall upward velocity above the slope. In a stationary state the equation governing the vertical density distribution is:

1 $$\begin{aligned} \partial _{z}(w\rho )-\partial _{z}(\kappa \partial _{z}\rho )=0. \end{aligned}$$

With a constant vertical velocity and diffusivity, this equation shows a linear relation between the density and its vertical gradient and has exponential solutions, with $\kappa /w$ as the e-folding length scale.

**Figure 4:**
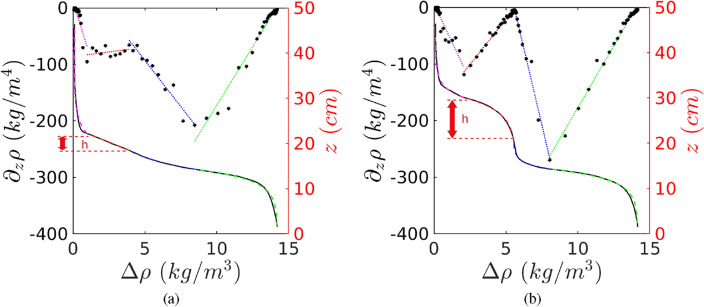
Same as Fig. 2a and c, with dashed lines representing piece-wise linear fits and continues lines the corresponding piece-wise exponential fits for $t/T=84$ (**a**) and $t/T=140$ (**b**) for Case 1. Red arrows in (**a**) and (**b**) show the thickness *h* measured from the vertical extension of the red exponential fit.

Figure 4a and b represents the profiles as given in Fig. 2a and c, fitted with four exponential functions whose boundaries are determined by the local extremes of the vertical density gradient: the transition from the upper layer to the intrusion (magenta), the intrusion (red), the transition to the bottom layer (blue) and the bottom layer (green). Solutions of Eq. (1) are calculated by imposing a starting vertical position $z_1$ and the corresponding density anomaly $\rho _1$ and stopping for $z_2<z_1$ and $\rho _2>\rho _1$, based on the four zones described here-above. These exponential fits are pertinent in each zone (see Fig. 4), here we focus on the intrusion zone. Note that the data disappears behind the piece-wise fit.

**Figure 5:**
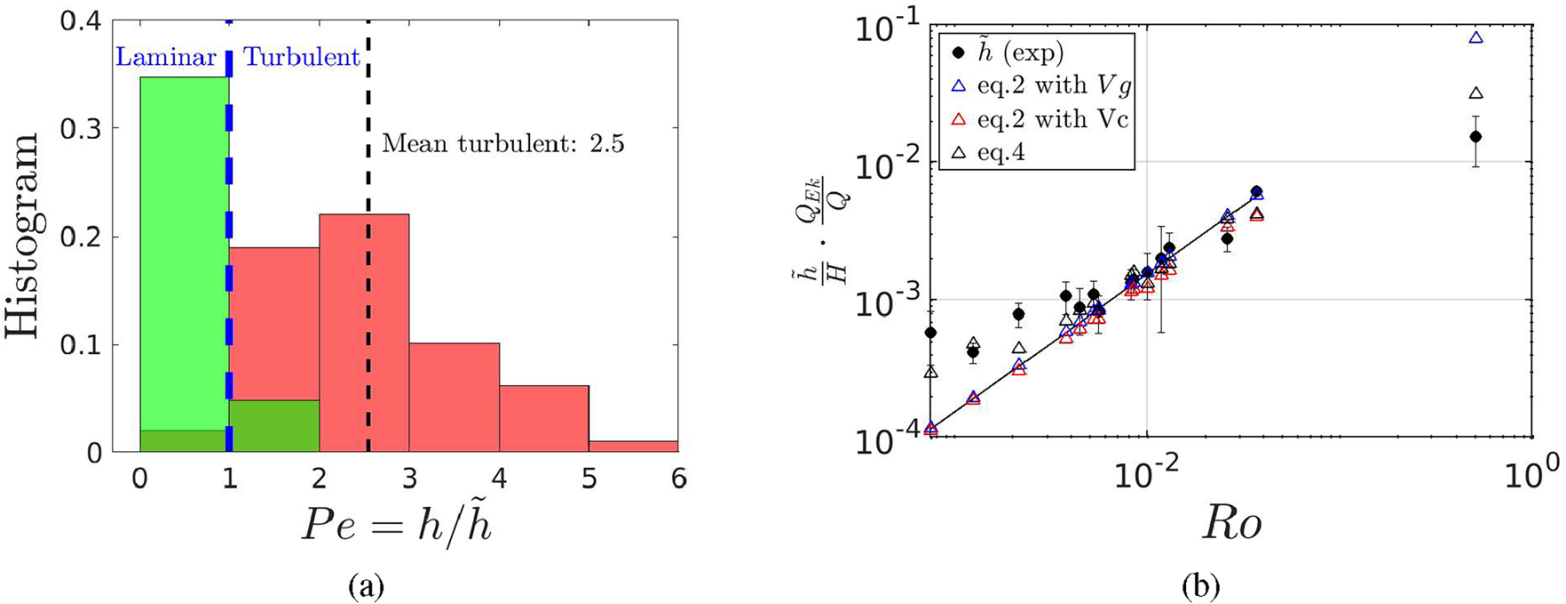
(**a**) Histogram of laminar and turbulent intrusions using the criteria $Pe\lessgtr 1$. The local maxima criteria is given in the same figure by green and red coloring. The averaged value for the turbulent intrusions is 2.5. (**b**) Measured scale height $\langle \tilde{h}\rangle$ obtained from averaged values of all the turbulent intrusions of a given experiment, for all the experiments (black dots) except for experiment Case 15 with a very low rotation ($Ro=0.51$), compared to the geostrophic model (blue triangles), geostrophic/cyclostrophic model with $R=4$ m (red triangles), geostrophic/cyclostrophic model and Poincaré waves with $k=10$ m. The continuous line represents a linear regression with a slope of $s/F^{2}$ and $F\approx 0.8$.

The exponential shape of the turbulent intrusion delivers the scale height $\tilde{h}=\kappa /w$^25^, which is the inverse of the e-folding coefficient. The thickness *h* of the intrusion is represented by the vertical extension *z* of the red line in Fig. 4a and b, determined as the vertical distance between the two extrema of the vertical density gradient of this portion of the density profile. Note that the ratio $h/\tilde{h}$ represents the Peclet number $Pe=Re_w Sc=h/\tilde{h}$, with $Re_w=wh/\nu$ and $Sc=\nu /\kappa$ and is a measure of the importance of diffusivity with respect to upward advection. If $Pe>1$, the upward advection *w* plays a significant role in the near-slope dynamics resulting in the observed exponential shape and the intrusion is turbulent. $Pe=h/\tilde{h}$ has an averaged value of 2.5 for turbulent intrusions, which demonstrates that $\tilde{h}$ is well defined. If $Pe<1$, the advective term becomes negligible compared to diffusion, giving rise to the observed linear shape and the intrusion is laminar. A histogram of all events for all the experiments considered is given in Fig. 5a. The local maxima criteria is given in the same figure by green and red coloring. The two independent criteria show a fair agreement. The vorticity sign criteria corroborates the observations from the $h/\tilde{h}$ criteria, with 84% of events with $\zeta <0$ corresponding to turbulent intrusions, and 82% of laminar intrusions corresponding to $\zeta >0$. We point out that $\tilde{h}$ is only meaningful in the case of turbulent intrusions where $\tilde{h}<h$, i.e. when $Pe>1$. In the next subsection we derive a theoretical model based on the critical Froude number which enables to predict $\tilde{h}$ as a function of the overall experimental parameters.

### Model for the scale height

To obtain an analytical expression for the scale height, the definition of the Froude number $F=V_{g}/\sqrt{g'\tilde{h}}$ can be used, where $V_{g}$ is the characteristic velocity scale which we first assume to be the geostrophic velocity. When the Froude number is subcritical a moving fluid layer can be laminar, when it becomes supercritical hydraulic jumps occur leading to turbulent dynamics. The turbulence leads to increased vertical mixing of density and momentum and thickens the moving layer until its Froude number becomes subcritical.

Setting the critical value $F=0.8$ for a rotating gravity current^27,28^, the scale height is given by

2 $$\begin{aligned} \tilde{h}=\frac{V_g^2}{g'F^2}=\frac{g'}{F^{2}f^{2}}s^2 \qquad \text {and in non-dimensional form} \qquad \frac{\tilde{h}}{H}=\frac{Q_{j}}{Q_{Ek}}Ro\frac{s}{F^{2}}, \end{aligned}$$

where $Q_{Ek}=2\pi R T_{Ek}$, with the intrusion radius $R=$4 m and $T_{Ek}=v_g \delta _E$ the Ekman transport and $\delta _E=\sqrt{2\nu /f}$. The Rossby number *Ro* is based on the horizontal scale $\mathscr {L}=Q_{j}/T_{Ek}$ with ${\mathscr {L}}$ representing the distance needed to drain the initial flux $Q_j$ through the Ekman layer, already defined by Lane-Serff and Baines^23^. Since the term $s/F^2$ is constant, the normalized relation in Eq. (2) states that the variation of the scale height $\tilde{h}$ is directly proportional to the ratio between the injected volume $Q_{j}$ and the total Ekman transport $Q_{Ek}$ multiplied by the Rossby number *Ro*. This proportionality can be observed in Fig. 5b, where the normalized and compensated scale heights are plotted as black symbols, for all the experiments. The experimental data (except Case 15 with a low rotation) can be fitted with a linear relation (continuous line) which slope corresponds to $s/F^2$ with $F=0.8$ as given in Eq. (2) and, consequently, in agreement with the literature^27,28^.

A more sophisticated model can be obtained taking into account the cyclostrophy, when the relative vorticity $\zeta$ becomes comparable to the planetary vorticity *f*. The effect of both geostrophy and cyclostrophy gives an alternative relation for the velocity scale, now $V_{c}$, to be included in Eq. (2) rather than $V_g$, which can be derived using the gradient balance^29^

3 $$\begin{aligned} fV_{c}+\frac{2V_{c}^{2}}{R}=g's. \end{aligned}$$

To further improve the prediction of $\tilde{h}$ for higher rotation rates, the effect of dispersive Poincaré waves should be considered, which replace shallow water waves in rotating flows. Their group velocity is given by $C_{PC}=\frac{g'\tilde{h}k}{\sqrt{f^{2}+g'\tilde{h}k^{2}}}$ (see^30^), to be used in a revised definition of the Froude number. This leads to

4 $$\begin{aligned} \tilde{h}=\frac{V_{c}^{2}}{2F^{2}g'}+\sqrt{\frac{V_{c}^4}{4g'^{2}F^{4}} +\frac{V_{c}^{2}f^{2}}{g'^{2}k^{2}F^{2}}}, \end{aligned}$$

where *k* is the horizontal wave number characteristic of the Poincaré waves which is estimated to be of the order $k={10}$ m^−1^.

These two revised models are plotted in Fig. 5b and compared to the purely geostrophic model using $V_g$ (blue symbols) and the experimental data (black circles). The purely geostrophic model fails to predict $\tilde{h}$ for a low rotation rate, whereas the cyclostrophic model [Eq. (2), red symbols] enables to improve the prediction. These models do not predict well the experimental data for high rotation rates. Instead, Eq. (4) (black triangles in Fig. 5b) well predicts the full range of experimental data over 3 decades of the Rossby number *Ro*.

### Interior or boundary mixing?

Topographic features are known to strongly increase the mixing in the deep ocean^31^, but quantitative estimates are scant. An implicit hypothesis made so far is that the turbulent fluxes occur in the gravity current phase and that the vertical density structure of the intrusion is conserved after the current has detached from the boundary, i.e. that no mixing occurs in the interior. In order to ascertain that no mixing takes place in the interior, we compute the Richardson number defined as:

5 $$\begin{aligned} Ri=-\frac{g}{\rho _{t}}\frac{\partial _{z}\rho }{(\partial _{z}U_{h})^{2}}=\frac{N^{2}}{(\partial _{z} U_{h})^{2}}, \end{aligned}$$

where $U_{h}$ is the horizontal component of the velocity and *N* the Brunt–Väissälä frequency. To combine the velocity and density measurement having different spatial and temporal resolutions, a discretised calculation of the Richardson number has been performed, using upward and central approximations for the density and horizontal velocity, respectively.

**Figure 6:**
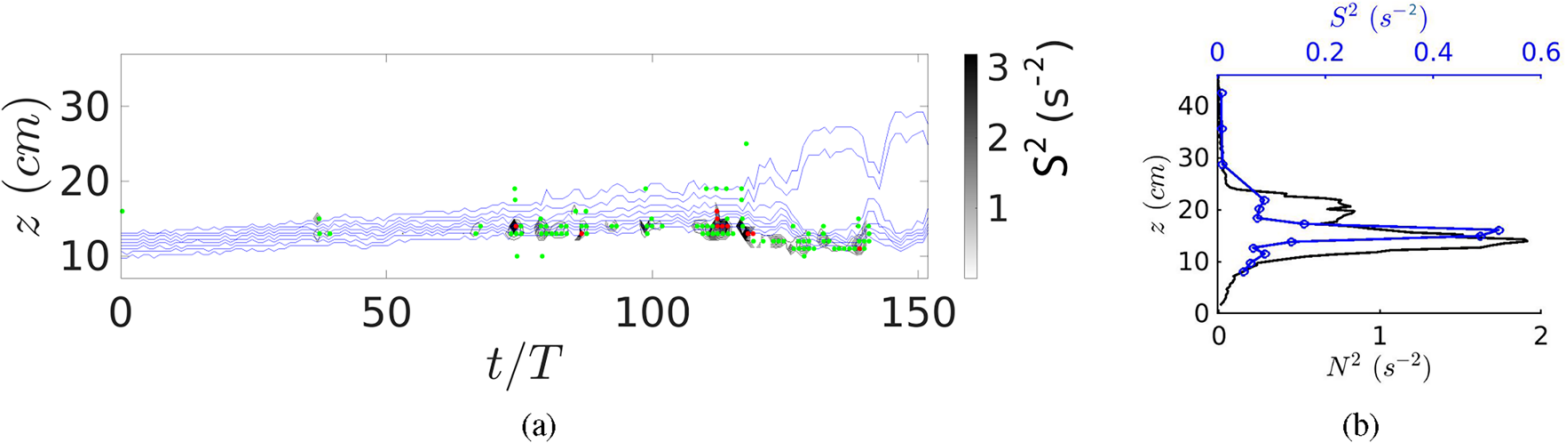
(**a**) Hovmöller diagram showing haloclines (blue lines) and the strain $S^2=(\partial _z U_h)^2$ concentrated at the lower boundary of the intrusion for Case 1. The green dots are relative to $1<Ri<10$ and the red dots to $0.8<Ri<1$. (**b**) For the same experiment, instantaneous vertical profiles of $N^2$ and $S^2$ (black and blue curves, respectively) at $t/T=84$ representative of a laminar intrusion.

The lowest Richardson numbers are located at the lower interface of the intrusion, but remain well above the critical condition $Ri=0.25$ for all the considered experiments. This can be seen in Fig. 6a where we superpose the temporal variation of the density anomaly (blue lines), the strain $S^2=(\partial _zU_h)^2$ and the locations where $1<Ri<10$ (green dots) and $0.8<Ri<1$ (red dots) for experiment Case 1. For the same experiment, the instantaneous vertical profiles of $N^2$ and $S^2$ (black and blue curves, respectively) at $t/T=84$, representative of a laminar intrusion, are plotted in Fig. 6b. The highest density gradients are located at the upper and lower boundaries of the intrusion layer. The strain $S^2$ has the largest value close to the bottom of the intruding layer, explaining the observed low values of the Richardson number in this region. Note that low Richardson numbers are not detected at the upper interface, neither for the laminar nor for the turbulent intrusions.

**Figure 7:**
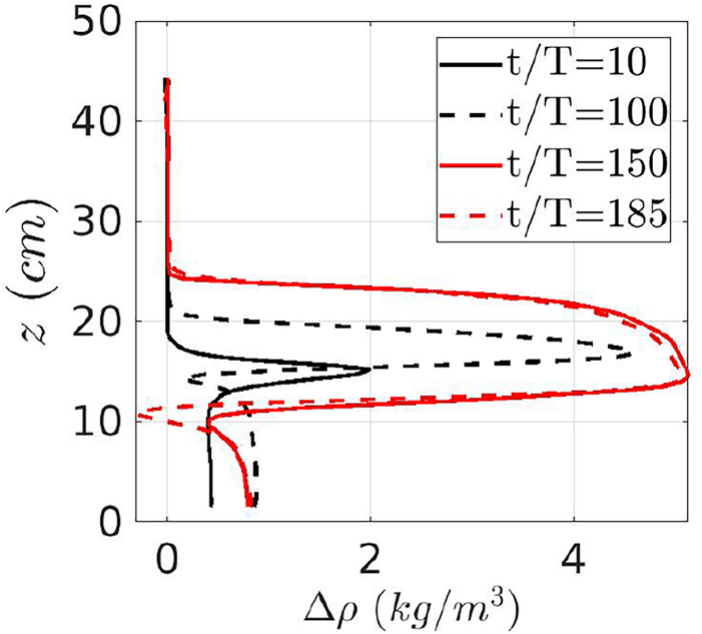
Vertical intrusion profiles for Case 10 with the injected density initially symmetric with respect to the densities of the top and bottom layers, plotted at different times for laminar (black lines) and turbulent (red lines) intrusions corrected by the measured halocline elevation. The laminar intrusions exhibit a symmetric shape, while the turbulent intrusions has a pronounced asymmetric shape.

To corroborate the fact that the saline water has mixed on the slope area rather than during the intruding phase in the ocean interior, mostly through dense water cascading, we consider in Fig. 7 the vertical density profile of the laminar (black curves) and turbulent (red curves) intrusions for experiment Case 10 at different times after the initial density structure has been subtracted. For this experiment, the injected density is exactly the average of the top and bottom densities ($\rho _{b}={1009}$ kg m^−3^, $\rho _{j}={1004.2}$ kg m^−3^, $\rho _{t}={999.6}$ kg m^−3^), and is hence initially symmetric. The vertical shape for laminar intrusions (black curves) is close to symmetric and this suggests that very low mixing happened on the slope. Turbulent intrusions, instead, present a pronounced asymmetry: a strong gradient is present at the lower boundary and a smoother gradient at the upper boundary. This is also in accord with observations^32^.

## Discussion and conclusions

Two distinct regimes for the intrusion of rotating gravity currents from the slope into the interior have been identified: laminar intrusions driven by Ekman dynamics and turbulent intrusions subject to turbulent mixing on the slope. The former gives rise to a local close-to-linear variation of the density with depth, whereas the latter leads to an exponential shape for the density structure of the intrusion. The computed Richardson number is well above the critical value of 0.25 demonstrating that no turbulent mixing takes place in the interior, but rather during the gravity current phase on the slope.

Using the diffusion–advection model^19^, the e-folding coefficient w/$\kappa$ of the exponential fits (also called the scale height) has been obtained, showing a balance between upward advection and downward diffusion of dense water. The two distinct intrusion regimes have been detected using the scale height: for laminar intrusions, the e-folding scale height w/$\kappa$ is small, the diffusivity is hence predominant over the upward velocity. For turbulent intrusions, a significant e-folding scale height underlines the influence of a strong upward advection *w* in the dynamics above the slope, resulting in the characteristic exponential shape. For turbulent intrusions, we derive a relationship which permits to determine the e-folding scale height from the variables *s*, *Ro*, $Q_{j}$ and $T_{Ek}$ which are set from the initial experimental parameters.

The e-folding scale height delivers a criteria to discriminate between turbulent and laminar intrusions: the intrusion is laminar when $h/\tilde{h}<1$, whereas it is turbulent when $h/\tilde{h}>1$ and $\tilde{h}$ is well defined. Further criteria based on the local maxima of the vertical density gradient and on the vorticity sign corroborate the results obtained with the first criteria. For turbulent intrusions we found that the thickness of the current *h* is, in average, 2.5 times the scale height $\tilde{h}$. An analytical model for predicting the scale height $\tilde{h}$ has been derived, which is able to predict its value over three decades of the Rossby number.

The motivation for this work was to understand the dynamics of intruding dense water on slopes in the ocean and we compare how this work relates to observations. The external parameters of the experiment where chosen so that the relevant non-dimensional parameters cover a wide range of scales so that they are representative of a large variety of oceanic intruding gravity currents, e.g. the downslope flow dominating as the Adriatic Outflow into the Ionian Sea^33^ and the rotation dominating as the Gibraltar outflow^34^. The similarity could not be achieved for the Reynolds number, which is many orders of magnitude larger in the ocean, although the size of the Coriolis platform allows for turbulent dynamics. We first consider observational data relative of the intrusion of dense water into the Weddell Sea. Recent investigations conducted between 2017 and 2019, and analyzed in 2023, have determined the thickness of a gravity current to about 150 m^9,35^. For a slope of $3.2 \times 10^{-2}$ (typical for the Weddell Sea), our model predicts a scale height of $\tilde{h}\approx$ 45 m which translates to a thickness of $h\approx \tilde{h}\cdot 2.5\approx$ 112.5 m. Intruding gravity currents of Mediterranean water at the Strait of Gibraltar, lead to intrusions further downstream giving rise to anti-cyclonic Mediterranean Eddies (Meddies). In 1992, Meddy Bobby was discovered southeast of the Azores, far from the Strait of Gibraltar, at approximately 34.75° N, 23.5° W. Its water properties, density structure, and velocity field have been studied by Pingree and Le Cann^36^. They suggest that Meddy Bobby remained relatively stable during its journey, with only minor mixing observed at its edges. With a radius of about 30 km, its vertical structure (cf. Fig. 9 in^36^) shows a dense water mass moving anti-cyclonically, with isopycnals condensed at the lower surface which is in accord with what we observed. Its thickness of about 900 m suggests, using our model, a slope of $1.5\times 10^{-2}$, which is typical for the shelf off-shore of the Portuguese coast where the gravity current evolves before it intrudes into the interior. Note that in this case of a rapidly rotating Meddy, the cyclostrophy dominates over geostrophy. The results shows clearly that our model enables to connect directly laboratory experiments and deep sea observations, and emphasizes the importance of laboratory experiments in understanding climate dynamics.

## Data Availability

All data used is available upon request (eletta.negretti@legi.cnrs.fr, LEGI/CORIOLIS).
